# Molecular evidence of the inhibitory potential of melatonin against sodium arsenite toxicity

**DOI:** 10.1016/j.heliyon.2025.e42113

**Published:** 2025-01-21

**Authors:** Ramtin Farhadi, Marzieh Daniali, Maryam Baeeri, Reza Khorasani, Hamed Haghi-Aminjan, Mahdi Gholami, Mahban Rahimifard, Mona Navaei-Nigjeh, Mohammad Abdollahi

**Affiliations:** a Department of Toxicology and Pharmacology, Faculty of Pharmacy, Tehran University of Medical Sciences (TUMS), Tehran, Iran; b Toxicology and Diseases Specialty Group, Pharmaceutical Sciences Research Center (PSRC), Tehran University of Medical Sciences (TUMS), Tehran, Iran

**Keywords:** Sodium arsenite, Melatonin, Oxidative stress, Inflammation, Diabetes

## Abstract

**Introduction:**

Sodium arsenite (SA), NaAsO2, is among the most hazardous toxicants, and wide use and presence of this toxicant leads to a severe environmental threat. Exposure to SA is associated with many health concerns, such as the prevalence of cancer and diabetes mellitus type 2 (DMT2). Many studies suggest that SA induces inflammation and biochemical impairments through different mechanisms, including increasing oxidative stress and altering vital genes such as biochemical and anti-inflammatory. Recent studies on melatonin (MLT), a harmless hormone secreted in the body generally for induction of sleepiness, find many beneficial and positive effects. Mitigating different harms and toxicities through different mechanisms, such as antioxidant properties, anti-inflammatory effects, and critical gene regulation, is essential. Due to these findings, this study aimed to evaluate the hypothesis that MLT may ameliorate pancreatic damage caused by exposure to SA.

**Methods:**

Forty-eight adult healthy male wistar rats aged 7–8 weeks were divided into eight for this research. Group 1 did not receive any intervention. Group 2 received 10 mg/kg/day MLT through intraperitoneal (IP) injection. Groups 3, 4, and 5 received 1.5 (1/10 LD50), 5 (1/3 LD50), and 7.5 (1/2 LD50) mg/kg SA, respectively. Groups 6, 7, and 8 were given 1.5 (1/10 LD50), 5 (1/3 LD50), and 7.5 (1/2 LD50) mg/kg of SA along with 10 mg/kg/day MLT, respectively, during the last ten days of the experiment. After 28 days of the experiment, the blood and tissue samples of the pancreas were removed for biochemical and pathological examination.

**Results:**

MLT attenuates SA toxicity by reducing oxidative stress biomarkers and inflammation markers. Moreover, MLT improves SA exposure's biochemical and functional damages by regulating related genes and pathways.

**Conclusion:**

MLT poses protective and preventive effects on the pancreas against exposure to SA. However, MLT's therapeutic and beneficial impacts have great potential for further investigation.

## 1 Introduction

Arsenic contamination in drinking water represents a significant global public health concern, affecting millions of people worldwide through both natural and anthropogenic sources. This toxic metalloid, primarily occurring in two forms - arsenite (As³⁺) and arsenate (As⁵⁺) - enters drinking water through various pathways, including natural geological processes, mining activities, industrial discharge, and agricultural runoff. The toxicity of arsenic operates through multiple mechanisms at the cellular level, primarily by disrupting ATP production, generating harmful reactive oxygen species, and interfering with DNA repair mechanisms and protein synthesis. Chronic exposure to arsenic-contaminated drinking water can lead to severe health consequences, including various types of cancer (particularly skin, bladder, and lung), cardiovascular diseases, neurological disorders, and diabetes. This problem is particularly acute in regions such as Bangladesh, West Bengal (India), and parts of South America, where geological conditions and limited access to water treatment technologies exacerbate the issue. While several effective treatment methods exist, including oxidation/filtration, ion exchange, and reverse osmosis, the implementation of these solutions often faces challenges due to economic and infrastructural limitations in affected regions, making arsenic contamination an ongoing challenge in global water quality management [[1], [2], [3], [4], [5], [6], [7]]. There are several mechanisms by which arsenic affects human health. One of the most critical underlying mechanisms for heavy metal toxicities such as arsenic is oxidative stress and the generation of reactive oxygen species (ROS) [1,8,9]. Furthermore, it is more than a hundred years since arsenic is known to be a carcinogen. Different studies showed that exposure to arsenic, either by digestion or inhalation, increases the risk of developing skin, bladder, liver, and lung cancers [10,11]. Besides, arsenic exposure is also associated with type II diabetes mellitus [12] and cardiovascular diseases [13]. In the pancreas, by altering the essential insulin-related genes such as Glut2 and Pdx1, arsenic results in a higher risk of diabetes mellitus type 2 (DMT2) and blood sugar dysregulation [14,15]. A recent study also showed that ROS itself is related to DMT2 incidence through DNA damage [16]. Inflammation is another underlying mechanism of arsenic toxicity that can even result in different diseases and cancer. Inflammation comes with the alteration of related factors, including proinflammatory cytokines (such as TNF-α, NF-κB) and interleukins (such as IL-6) [17,18]. Also, two studies were conducted recently and investigated the effects of arsenic on the liver and cardiopulmonary organs. These studies showed that arsenic poses toxic effects through the alteration in the Sirt1/Nrf2/TNF-α signaling pathway, which may be ameliorated by an agent that positively affects this pathway [19,20].

Melatonin (MLT) is a powerful endogenous antioxidant hormone, playing a crucial role in attenuating oxidative stress through multiple mechanisms [21]. MLT is known for its role in circadian regulation, is a hormone the pineal gland usually secures. Recent studies revealed antioxidant, anti-inflammatory, immunomodulatory, hemostasis, and glucose regulation effects of MLT [[22], [23], [24]]. Furthermore, MLT brings antioxidant effects by scavenging reactive oxygen species (ROS) through lipid peroxidation inhibition [25,26]. One of the most common pathways related to pancreatitis is throughout the Sirt1/Nrf2 pathway, which is ameliorated in the neurologic system by the MLT effects [[27], [28]].

These modulating effects of MLT bring the idea that exogenous MLT could be one of the safest solutions against arsenic toxicity and its problems in people who are dangerously at risk of exposure. So, in this study, we investigated the ameliorating effects of MLT on arsenic toxicity in the pancreas.

## 2 Materials and methods

### 2.1 Chemicals

MLT (CAS number 73-31-4), NaAsO2 (CAS number 7784-46-5), and all the other chemicals used in this study were obtained from Sigma-Aldrich (Munich, Germany). In addition, the Ribonucleic acid (RNA) extraction kit and cDNA synthesis kit for gene expression experiments were obtained from Sacace® (Como, Italy) and ThermoScientific® (Waltham, MA, USA), respectively. Also, insulin was measured using a Micromedia® (Uppsala, Sweden) kit. A blood glucose glucometer named Accu-check was obtained from Roche® (Indianapolis, Indiana).

### 2.2 Ethical approval

All steps of this study in association with animals, such as keeping, treating animals, sacrificing, and all the other procedures, were conducted according to the regulations regarding animal experiments. This study received its ethical approval code from the Tehran University of Medical Science (TUMS) under the IR code. TUMS.MEDICINE.REC.1400.570. Furthermore, all biochemical experiments and laboratory procedures were done according to Good Laboratory Practices.

### 2.3 Animals

Male wistar rats were chosen for this study over female rats due to the impact of cyclic variations in the level of female hormones on toxicological studies. Thus, disease-free, healthy, and 7 to 8-week-old male Wistar rats weighing between 170 and 180 g were obtained from the animal house of the Faculty of Pharmacy of Tehran University of Medical Sciences, Tehran, Iran. Rats were moved to the laboratory two weeks before the experiment for environmental adaptation. Environment standard conditions were provided, including room temperature of 25 ± 1 °C, 12-h light and dark period, and relative humidity of about 50–55 %. Also, free and reasonably equivalent access to a standard diet and water was provided for all of the groups.

### 2.4 Study design

In this study, solutions of Sodium Arsenite (SA) and MLT were prepared daily to be fresh. Both SA and MLT were dissolved in normal saline (NS) with the proper concentrations and administered with suitable doses. Dose of melatonin, SA lethal dose 50 (LD50), and three portion of the LD50 were chosen from previous studies [29,30]. Rats were divided into eight groups of six rats each. This study was designed as a sub-acute model of SA toxicity and amelioration with MLT in the following manner:

Group 1 had a normal diet and did not receive anything.

Group 2 received an intraperitoneal (IP) injection of MLT with a 10 mg/kg/day dose in the last ten days of the study.

Groups 3, 4, and 5 received 1.5 (1/10 LD50), 5 (1/3 LD50), and 7.5 (1/2 LD50) mg/kg of SA, respectively.

Groups 6, 7, and 8 were given 1.5 (1/10 LD50), 5 (1/3 LD50), and 7.5 (1/2 LD50) mg/kg of SA, respectively too, with the difference that these groups during the last ten days of the experiment treated with 10 mg/kg/day of MLT.

**Fig. 1:**
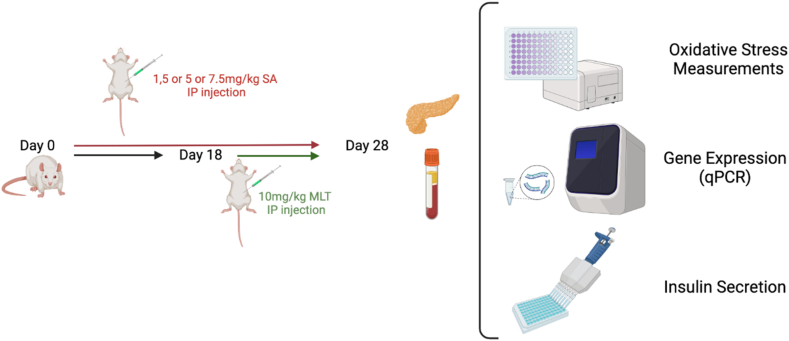
Summary of the experiment and study design. Healthy male wistar rats aged 7–8 weeks were divided into eight groups of 6 rats. Group 1 received normal saline intraperitoneally. Group 2 received an intraperitoneal (IP) injection of 10 mg/kg/day MLT for the last ten days of the experiment. Groups 3, 4, and 5 received 1.5 (1/10 LD50), 5 (1/3 LD50), and 7.5 (1/2 LD50) mg/kg SA, respectively, for 30 days to induce sub-acute toxicity. Groups 6, 7, and 8 were treated just the same as groups 3, 4, and 5, with the difference of getting 10 mg/kg/day of MLT for the last ten days of the experiment. After 28 days of treatment, pancreas tissues and blood samples were isolated for further biochemical and genetic experiments.

After 28 days of treatment, rats were sacrificed with IP injections of 100 mg/kg of ketamine and 10 mg/kg of xylazine. Ethylene diamine tetra acetic acid (EDTA) and serum separator tubes were used to collect blood samples. Pancreases were removed and frozen at −80 °C for further biochemical analysis immediately. An overview of the study is summarized in Fig. 1.

### 2.5 Oxidative stress markers

100 mg of the pancreas samples were homogenized with phosphate buffer (PBS). The homogenized mixture was centrifuged for 5 min at 3000 g. The supernatants obtained from centrifuging were collected to investigate the amount of oxidative stress biomarkers.

### 2.6 Determination of ROS level

The electron transportation activity of mitochondria produces ROS as a main by-product [31]. In biological and low concentrations, it is one of the most vital and regulating factors in cell pathways. ROS overproduction and high levels of that induce many side effects such as damage, necrosis, apoptosis, and many others [32]. For these reasons, in cases of investigations on toxicity and damages, ROS could be an essential factor to be measured due to the following protocol. There are different biomarkers to be measured for the determination of ROS. The most important and common is Dichlorodihydrofluorescein diacetate (DCFH-DA) [33]. To measure ROS, 5 μL of DCFH-DA was added to 25 μL supernatant of the homogenized tissues along with 81 μL of assay buffer. The mixture was incubated for 15 min at 37 °C in the next step. Lastly, the fluorometric absorbance of the samples was measured at the 485 nm wavelength for 60 min.

ROS level is reported in mol/min/mg protein unit. This unit form is a result of making ROS levels comparable. In order to make it comparable, it should be normalized with the amount of tissue's protein first. To do that, protein level was measured according to the Bradford Protein Assay (BPA). First, 100 μL of Bradford reagent was added to 10 μL of each sample. Then, the mixture was put in a dark place for 30 min. Moreover, for the last step, the protein amount was determined by measuring Fluorometric absorbance at the wavelength of 595 nm. Finally, we reported the amount in the mentioned unit which is mol/min/mg protein unit.

### 2.7 Quantitative real-time reverse transcription PCR for gene expression

Investigating gene expressions, especially those that have vital effects on the body or help the body ameliorate after damage, is essential in toxicology studies. For this purpose in this study, quantitative real-time reverse transcription polymerase chain reaction (qRT-PCR) was used. Two different classifications of genes, including inflammatory and aging genes (such as *TNF-α*, *NF-κB*, and *P53*) and also biochemistry-related genes (such as *Sirt1*, *Pdx1*, and *UCP2*) were chosen to be investigated in this respect. Firstly, frozen tissue samples' RNA was extracted using the Sacace® ribonucleic acid extraction Kit protocol. Thermo Scientific NanoDrop (Thermo Scientific, USA) was utilized to check RNA extraction purity and concentration. To make RNA more pure, genomic deoxyribonucleic acid (DNA) has to be removed. For this purpose, the RNase-Free DNase I Kit was used. Following, 1 μg of this pure RNA was reverse-transcribed to cDNA due to ThermoScientific® the kit protocol. The glyceraldehyde 3-phosphate dehydrogenase (*GAPDH*) gene, a housekeeping gene, was chosen to be an internal control to normalize the expression of the genes of interest in proportion to the expression of this gene and make them comparable. The LightCycler® 96 System (Roche, USA) is the equipment that was utilized for qRT-PCR analysis in our experiment. They relative gene expression were reported using Pfaffle Method (standard curve and ΔΔCP methods) [34].

The designed primers utilized in this study for each gene are shown in Table 1.

**Table 1:** Sequences of the primers used to assess gene expressions in RT-PCR assay.

Gene name	Gene symbol	Accession no.	Primer sequence (5′-3′)
Glyceraldehyde-3 phosphate dehydrogenase	*GAPDH*	NM-017008.4	F:ACTCACGGCAAATTCAACGG
			R:GACACCAGTAGACTCCACGA
Tumor protein P53	*P38α*	NM-031020.2	F: GACACCCCCTGCTTATCTCA
			R: TATCCGAGTCCAAAACCAGC
Pancreatic and duodenal homeobox 1	*Pdx1*	NM_022852.3	F:CTCCGGACATCTCCCCATAC
			R:TTTCATCCACGGGAAAGGGA
Nuclear factor kappa B submit	*NF-κB*	NM-001276711.1	F: TTCAACATGGCAGACGACGA
			R: AGGTATGGGCCATCTGTTG
Nuclear factor erythroid 2-related factor 2	*Nrf2*	XM_006234396.2	F:TCCATTTACGGAGACCCACC
			R: GGCCTTCTGTTTGACACTT
Sirtuin 1	*SIRT1*	NM_019812.3	F:GGCTAGGTGGTGAATATGCCA
			R:CCAATTCCTTTTGTGGGCGT
Uncoupling Protein 2	*UCP2*	NM_011671.5	F:ATGTGGTAAAGGTCCGCTTC
			R: TGGTCTTGTAGGCTTCGACA
Tumor necrosis factor	*TNF-α*	NM_001278601.1	F: AGCCGATGGGTTGTACCTTG
			R:AGATAGCAAATCGGCTGACG
Interleukin 6	*IL-6*	NM_001278601.1	F: CGATACAGGTGATGATGATG
			R: TACTACCAGAGTGTCTAGGA
Glucose transporter 2	*GLUT2*	NM_031197.2	F: GCCCAGCAGTTCTCAGGAAT
			R: ACATGCCAATCATCCCGGTT

### 2.8 Statistical analysis

The results of this study were presented as Mean ± standard error of means (SEM). Each experiment was repeated at least twice, with six replicates. One-way analysis of variance (ANOVA) and Tukey's multiple-comparison tests were used to assess statistical differences. The significance of the changes was reported and set at P < 0.05.

## 3 Results

### 3.1 Oxidative stress biomarkers

#### 3.1.1 ROS

These species are natural by-products of cells and are beneficial in biological concentration [35]. Different issues, such as inflammation and upregulation of ROS concentration, bring about several toxic impacts involving cellular and genomic damage or dysfunction [36]. Therefore, ROS could be counted as a factor of damage and intoxication. ROS levels of pancreas tissue were assessed following administration of the three doses of SA, alone or in combination with MLT. Compared with the control group, treating rats with MLT in the absence of SA did not change ROS level statistically*,* indicating the safety profile of MLT administration.

**Fig. 2:**
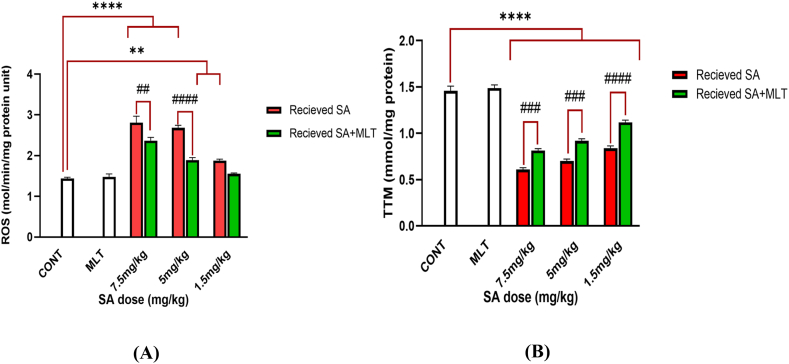
Assessment of ROS and TTM in Wistar rats exposed to MLT and SA. Results are expressed as mean ± SEM for six rats in each group (n = 6). ∗∗∗∗*p-value*<0.0001; ####*p-value*<0.0001. Fig. 2A. In ROS-related figure: ∗∗p-value = 0.0055 (Vs. control groups), ##p-value = 0.0055 (each group Vs. its Corresponding group). Fig. 2B. In TTM-related figure: ###*p-value* = 0.0002 (each group Vs. its Corresponding group). The control group received an IP injection of normal saline for 28 days, melatonin group received an IP injection of melatonin with 10 mg/kg dose during the last ten days of the study, in each corresponding pair of 7.5 mg/kg, 5 mg/kg, and 1.5 mg/kg groups there are two groups; one which is shown with red graph means receiving IP injection of SA with the mentioned dose for 28 days, and the other one is shown with green graph which means receiving IP injection of SA with the mentioned dose for 28 days too, but with the difference that during the last ten days of the study, they received IP injection of melatonin with 10 mg/kg dose to investigate the effects of melatonin on SA toxicity. Abbreviations: ROS: reactive oxygen species; TTM: total thiol group SA: sodium arsenite; MLT: melatonin; CONT: Control group.

On the other hand, there was a significant increase in ROS levels in the groups that received SA, compared to the control groups (*p-value*<0.0001), which shows the impact of SA in inducing ROS. The ameliorating effects of MLT are shown in each SA group dose. ROS level in groups that received SA with the dose of 7.5 mg/kg and 5 mg/kg was reversed significantly when treated with MLT (in order *p-value*<0.01 and *p-value*<0.0001). The group that received 1.5 mg/kg SA alone showed a significant increase in ROS level compared to the control group. Interestingly, when treated with MLT, this group shows no significant difference from the control group. These results, shown in Fig. 2A, indicate that treatment with MLT could reverse the increase in ROS levels caused by SA.

#### 3.1.2 Total thiol group (TTM)

TTM is another factor whose reduction indicates oxidative stress-induced cellular damage [37]. Treating rats with MLT in the absence of SA did not change ROS levels significantly, indicating the safety profile of MLT administration. On the other hand, as shown in Fig. 2B, receiving SA in the six other groups reduces TTM significantly (*p-value*<0.0001). Fig. 2B also shows the effect of MLT on receiving SA statically. All three doses of SA, when followed by treatment of MLT, showed a significant increase in TTM level. To explain in details, receiving SA alone with the dose of 7.5 mg/kg compared to receiving it with MLT treatment has a significamt different with *p-value*<0.001. Also, receiving 5 mg/kg and 1.5 mg/kg doses alone show difference in comparison with receiving them with following MLT treatment with a *p-value*<0.0001.

### 3.2 Gene expression analysis

#### 3.2.1 Inflammatory cytokines gene expression

##### 3.2.1.1 TNF-α gene expression

**Fig. 3:**
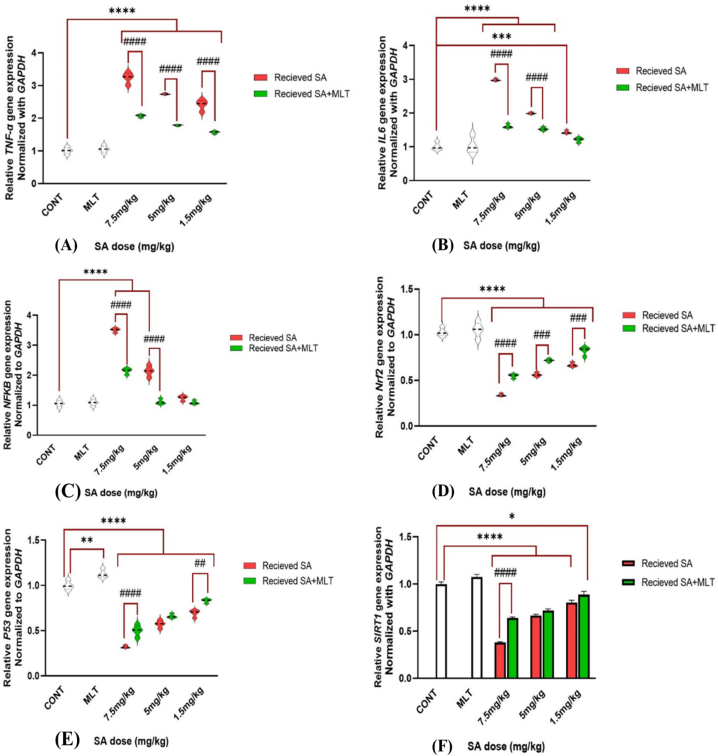
A, 3B, 3C, 3D, 3E, and 3F. Assessment of inflammation-related gene expression in Wistar rats exposed to MLT and SA. Results are expressed as mean ± SEM for six rats in each group (n = 6). ∗∗∗∗*p-value*<0.0001 (Vs. control groups), ####*p-value*<0.0001 (each group Vs. its Corresponding group). In IL-6 figure (B): ∗∗∗*p-value* = 0.0001 (Vs. control groups). In Nrf2 figure (D): ###*p-value* = 0.0002 (each group Vs. its Corresponding group). In P53 figure (E): ∗∗*p-value* = 0.0026 (Vs. control groups), ##*p-value* = 0.0012 (each group Vs. its Corresponding group). In SIRT1 figure (F): ∗*p-value* = 0.0289 (Vs. control groups). The control group received an IP injection of normal saline for 28 days, melatonin group received an IP injection of melatonin with a 10 mg/kg dose during the last ten days of the study, in each corresponding pair of 7.5 mg/kg, 5 mg/kg, and 1.5 mg/kg groups there are two groups; one which is shown with red graph means receiving IP injection of SA with the mentioned dose for 28 days, and the other one is shown with green graph which means receiving IP injection of SA with the mentioned dose for 28 days too, but with the difference that during the last ten days of the study, they received IP injection of melatonin with 10 mg/kg dose to investigate the effects of melatonin on SA toxicity. Abbreviations: SA: sodium arsenite; MLT: melatonin; CONT: Control group.

TNF-α is an immune system mediator released from macrophages in response to external or internal pathogens. Although TNF-α must be released to help the body resist exposure to toxicants and pathogens, it has some adverse effects, too. It causes inflammation and cellular dysfunction [38]. Measuring *TNF-α* gene expression of the different groups in proportion to the control group of this study is demonstrated in Fig. 3A. The results indicate that receiving MLT does not increase the *TNF-α* gene expression*,* which means the safety profile of MLT administration.

Compared to the control group, receiving SA with doses of 7.5 mg/kg, 5 mg/kg, and 1.5 mg/kg causes a significantly increased *TNF-α* gene expression (*p-value*<0.0001). As shown in Fig. 3A, results show that MLT can significantly reverse the *TNF-α* gene expression increase caused by every dose of SA administration (*p-value*<0.0001). These findings confirm the potential role of MLT in SA toxicity amelioration.

##### 3.2.1.2 IL-6 gene expression

IL-6 increases when the body faces infections, toxicities, or other attacks and damages. So, an increase in that is a factor of inflammation, damage, and toxicity. Although IL-6 helps the body fight, chronic release causes inflammation-associated disease and even autoimmune disease [39,40]. Fig. 3B demonstrates the proportion of *IL-6* gene expression of the different groups to the control group of this study. The results indicate that there is not any significant increase in *IL-6* gene expression in the group receiving MLT*,* which shows the safety profile of MLT.

In comparison to the control group, receiving SA increases *IL-6* gene expression dose-dependently (Groups that received 7.5 mg/kg and 5 mg/kg of SA have *p-value*<0.0001, but the group that received 1.5 mg/kg does not show any significant change in *IL-6* gene expression). As evident in Fig. 3B, MLT reverses the increase of *IL-6* gene expression very effectively (*p-value*<0.0001), which can inhibit the toxicity of the SA. These findings confirm the potential role of MLT in SA toxicity amelioration.

##### 3.2.1.3 NFkB gene expression

Another factor that increases signs of inflammation and toxicity is NFkB, and measuring NFkB expression levels is critical for investigating inflammation, damage, and toxicity. Also, recent studies showed that despite the positive effects of NFkB on multiple aspects of immune functions and inflammatory responses, it has adverse effects in developing cancer and inflammation-based disease [41,42]. Fig. 3C demonstrates the proportion of *NFkB* gene expression of the different groups to the control group of this study. The results indicate that there is not any significant increase in *NFkB* gene expression in the group receiving MLT*,* which shows the safety profile of MLT. Compared to the control group, receiving SA increases *NFkB* gene expression dose-dependently (Groups that received 7.5 mg/kg and 5 mg/kg of SA have *p-value*<0.0001, but the group that received 1.5 mg/kg does not show any significant changes). As evident in Fig. 3C, MLT reverses the increase of *NFkB* gene expression very effectively (*p-value*<0.0001), which can inhibit the toxicity of the SA. Interestingly, the group that received 5 mg/kg SA did not show a meaningful difference compared with the control group after being treated with MLT. These findings indicate the potential role of MLT in SA toxicity amelioration.

##### 3.2.1.4 Nrf2 gene expression

One critical factor that regulates and activates antioxidant and protective pathways is nuclear factor erythroid 2–related factor 2 (Nrf2). Nrf2 and its related pathways fight against oxidative stress, inflammation, and toxicity via different pathways and mechanisms [43,44]. Because of the importance of Nrf2, we measured different groups of Nrf2 expression in proportion to the control group and brought that to Fig. 3D. According to that, it is clear that the administration of MLT did not reduce *Nrf2* expression compared with the control group, which shows the safety profile of MLT. Furthermore, it shows that receiving SA with any doses reduces *Nrf2* expression (*p-value*<0.0001). However, groups that received SA and were treated with MLT show a significant increase in *Nrf2* expression in comparison with receiving SA alone with that dose (Receiving SA alone with the dose of 7.5 mg/kg in comparison with receiving it with MLT treatment has *p-value*<0.0001 and receiving 5 mg/kg and 1.5 mg/kg doses alone in comparison with receiving them with following MLT treatment has *p-value*<0.001).

##### 3.2.1.5 P53 gene expression

Many recent studies are interested in P53 due to its pivotal and novel roles, such as reducing inflammation and some related factors (such as IL-6 and NFkB), suppressing DNA damages, reducing death of the cells, and aging [[45], [46], [47], [48]]. So, investigating the changes in P53 expression could be helpful in toxicology studies. Something interesting happened in the group that received MLT alone. As demonstrated in Fig. 3E, MLT did not reduce *P53* expression compared to the control group, and its level had been raised meaningfully compared to the control group (*p-value*<0.01). *P53* expression was reduced in the other groups that received SA compared to the control group (*p-value*<0.0001). Moreover, MLT reversed SA adverse effects on *P53* expression in the groups that received SA with 7.5 mg/kg and 1.5 mg/kg doses (in order *p-value*<0.0001 and *p-value*<0.01). These findings indicate the beneficial effects of MLT on SA toxicity and p53 expression.

##### 3.2.1.6 SIRT1 gene expression

Sirtuin 1 (SIRT1) is essential in inflammation regulation and biochemical pathways. It helps the body regulate oxidative stress and inflammation caused by toxicant damage in collaboration with Nrf2 pathways [49]. Moreover, SIRT1 helps the pancreas through different mechanisms, such as repressing Uncoupling protein 2 (*UCP2*) in pancreatic β cells and protecting β cells [50,51]. Therefore, *SIRT1* expression was measured in this study to investigate whether or not SA toxicity can pose adverse effects through this gene. Fig. 3F demonstrates that *SIRT1* expression in the group receiving MLT has no meaningful difference from the control group, showing MLT administration's safety.

On the other hand, each group exposed to any SA doses differs from the control group meaningfully (*p-value*<0.0001). The group that received SA with the dose of 7.5 mg/kg compared to the group that received SA with the same dose but followed by MLT treatment after that shows a significant difference, and MLT somehow reversed the adverse effects of SA. Furthermore, the group that received 1.5 mg/kg SA followed by MLT treatment shows a less meaningful difference from the control group compared to receiving the same dose of SA alone (*p-value*<0.05 versus *p-value*<0.0001). These findings suggest ameliorating the effects of MLT on SA toxicity.

#### 3.2.2 Functional gene expression, insulin secretion, and blood glucose level

##### 3.2.2.1 Pdx-1 gene expression

**Fig. 4:**
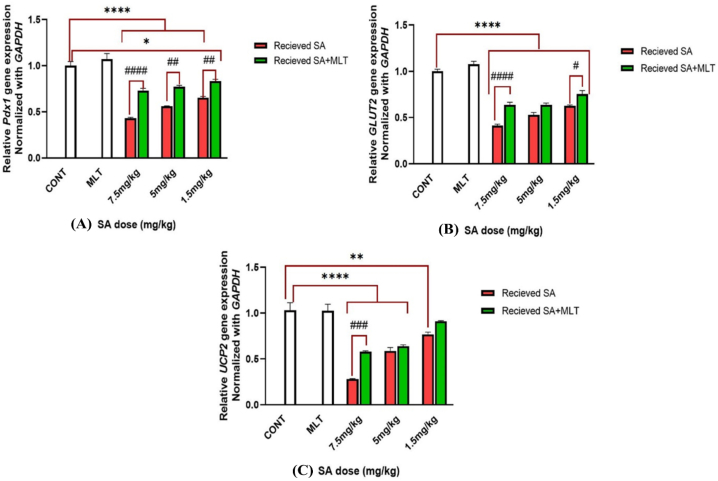
A, 4B, and 4C. Assessment of functional gene expression in Wistar rats exposed to MLT and SA. Results are expressed as mean ± SEM for six rats in each group (n = 6). ∗∗∗∗*p-value*<0.0001 (Vs. control groups), ####*p-value*<0.0001 (each group Vs. its Corresponding group). In Pdx1 figure (A): ∗*p-value* = 0.01 (Vs. control groups), ##*p-value* is equal to 0.001 and 0.0059 in order (each group Vs. its Corresponding group). In GLUT2 figure (B): #*p-value* = 0.03 (each group Vs. its Corresponding group). In UCP2 figure (C): ∗∗p-value = 0.0042 (Vs. control groups), ###p-value = 0.0009 (each group Vs. its Corresponding group). The control group received an IP injection of normal saline for 28 days, melatonin group received an IP injection of melatonin with a 10 mg/kg dose during the last ten days of the study, in each corresponding pair of 7.5 mg/kg, 5 mg/kg, and 1.5 mg/kg groups there are two groups; one which is shown with red graph means receiving IP injection of SA with the mentioned dose for 28 days, and the other one is shown with green graph which means receiving IP injection of SA with the mentioned dose for 28 days too, but with the difference that during the last ten days of the study, they received IP injection of melatonin with 10 mg/kg dose to investigate the effects of melatonin on SA toxicity. Abbreviations: SA: sodium arsenite; MLT: melatonin; CONT: Control group.

*The pdf-1* gene is the critical gene with the most vital role in the pancreas. This gene plays roles in the formation of all types of pancreatic cells, the development of islet β-cells, and the function of adult islet β-cells [52,53]. Due to its roles and regulatory effects on the other genes and pathways, it can be known as the essential gene related to the biochemical roles of the pancreas. According to Fig. 4A, the safety of MLT administration is apparent as Pdx-1 expression does not differ from that of the control group. Compared with the control group, receiving SA with any doses causes *Pdx-1* gene expression reduction significantly (*p-value*<0.0001). Different doses of SA groups showed *Pdx-1* reduction, which is reversed to some degrees after treatment with MLT (7.5 mg/kg, 5 mg/kg, and i.5 mg/kg dose of SA have *p-value*<0.0001, *p-value*<0.001 and *p-value*<0.01 in order). Furthermore, the group that received 1.5 mg/kg SA followed by MLT treatment shows a less meaningful difference from the control group compared to receiving the same dose of SA alone (*p-value*<0.01 versus *p-value*<0.0001). These findings suggest ameliorating the effects of MLT on SA toxicity.

##### 3.2.2.2 GLUT2 gene expression

GLUT2 facilitates glucose transport in different tissues and is critical in glucose sensing and homeostasis [54,55]. Different groups' *GLUT2* expressions in proportion to the control group are shown in Fig. 4B. The group receiving MLT did not differ from the control group, which means the safety profile of MLT administration. Administering SA with any doses reduces *GLUT2* expression (*p-value*<0.0001). However, treating groups receiving SA with 7.5 mg/kg and 1.5 mg/kg doses with MLT reverses the harmful effects of SA to some degree meaningfully (in order *p-value*<0.0001 and *p-value*<0.05). These findings suggest the healing effect of MLT on SA toxicity.

##### 3.2.2.3 UCP2 gene expression

Besides the oxidative stress regulatory effects of the UCP2, toxicants pose their harms through the down-regulation of *UCP2* and may lead to diabetes mellitus (DM) [56]. *UCP2* expression in the group receiving MLT has no difference from the control group statically, which means the safety profile of MLT administration; however, as shown in Fig. 4C, SA results in *UCP2* expression reduction dose-dependently (7.5 mg/kg and 5 mg/kg SA have *p-values* <0.0001 and 1.5 mg/kg *p-value*<0.01).

In the group receiving 7.5 mg/kg SA, MLT administration reversed the adverse effects of SA and upregulated *UCP2* to some degree (*p-value*<0.001).

##### 3.2.2.4 Blood insulin level analysis

**Fig. 5:**
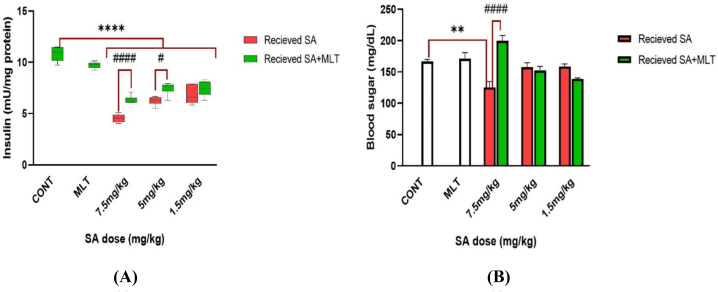
A and 5B. Assessment of insulin secretion level and blood sugar level in Wistar rats exposed to MLT and SA. Results are expressed as mean ± SEM for six rats in each group (n = 6). ∗∗∗∗*p-value*<0.0001 (Vs. control groups), ####*p-value*<0.0001 (each group Vs. its Corresponding group). In insulin figure (A): #*p-value* = 0.018 (each group Vs. its Corresponding group). In blood sugar figure (B): ∗∗*p-value* = 0.0069 (Vs. control groups). The control group received an IP injection of normal saline for 28 days, melatonin group received an IP injection of melatonin with 10 mg/kg dose during the last ten days of the study, in each corresponding pair of 7.5 mg/kg, 5 mg/kg, and 1.5 mg/kg groups there are two groups; one which is shown with red graph means receiving IP injection of SA with the mentioned dose for 28 days, and the other one is shown with green graph which means receiving IP injection of SA with the mentioned dose for 28 days too, but with the difference that during the last ten days of the study, they received IP injection of melatonin with 10 mg/kg dose to investigate the effects of melatonin on SA toxicity. Abbreviations: SA: sodium arsenite; MLT: melatonin; CONT: Control group.

As evident in Fig. 5A, compared to the control group, receiving MLT did not change the insulin level in the blood meaningfully, which means the safety profile of MLT administration. On the other hand, administration of SA reduces blood insulin meaningfully with any doses (*p-value*<0.0001). Furthermore, MLT shows its amelioration effects against SA toxicity in higher doses of SA. Insulin levels in the blood of the group receiving 1.5 mg/kg SA did not rise after treatment with MLT. However, in the groups receiving SA with 5 mg/kg and 7.5 mg/kg doses, MLT significantly reduced blood insulin (*p-value*<0.05 and *p-value*<0.0001).

##### 3.2.2.5 Blood sugar level analysis

Measuring blood sugar levels showed that receiving MLT alone and low doses of SA did not affect the blood sugar level compared to the control group. These results indicate the safety of the MLT administration. However, sub-acute exposure to the high dose of SA (7.5 mg/kg) results in hypoglycemia compared with the control group (*p-value*<0.01). The hypoglycemia effect of the 7.5 mg/kg SA is reversed by the administration of the MLT significantly (*p-value*<0.0001). These results are shown in Fig. 5B.

## 4 Discussion

Sodium arsenite, as one of the confirmed environmental factors in the prevalence of DM, is vital in the imbalance of oxidative stress biomarkers and inflammatory cytokines [29,57]. However, external MLT consumption shows beneficial impacts on different pancreatic inflammatory diseases. MLT can modulate various internal responses like immune and hormonal responses and is investigated in neutralizing ROS and reactive nitrogen species (RNS) [58,59].

In this study, MLT was used to show its protective impacts against the toxicity induced by three different concentrations of sodium arsenite. Blood glucose levels, expression of the GLUT2 gene, and secretion of insulin were improved by administering MLT to sodium-arsenite-received animals.

Also, the expression of inflammatory cytokines like IL-6 and TNF-α was significantly reduced when MLT was added to the sodium-arsenite regimen through the in-vivo section. Moreover, MLT showed its ability to enhance other cellular damaging conditions, too; administration of MLT among the animals exposed to sodium arsenite also demonstrated its beneficial impacts on oxidative stress like ROS and TTM and expression of *SIRT1*, *UCP2*, *NFkB*, and *P53*. Arsenic-induced oxidative stress triggers a complex cascade of molecular events involving key regulatory genes and inflammatory mediators. Nrf2, a master regulator of the antioxidant and infllamatory responses, becomes activated in response to arsenic-generated ROS, orchestrating the transcription of various cytoprotective genes [60,61]. Simultaneously, SIRT1, a crucial metabolic sensor and longevity-associated protein, works to suppress inflammatory responses and enhance cellular stress resistance [62], while also modulating UCP2, which helps regulate mitochondrial ROS production and energy metabolism [63]. However, persistent arsenic exposure leads to the activation of pro-inflammatory pathways, resulting in increased expression of TNF and IL -6, which further exacerbate cellular damage through inflammatory responses [[64], [65], [66]]. This inflammatory surge creates a vicious cycle where increased ROS production leads to enhanced inflammation, which in turn generates more ROS, potentially overwhelming the protective mechanisms of Nrf2 and SIRT1. These molecular interactions highlight the intricate balance between cellular defense mechanisms and inflammatory responses in arsenic toxicity, emphasizing the importance of therapeutic interventions that can modulate these pathways effectively.

Different studies confirmed the role of sodium arsenite in the prevalence of DM through in-vivo and in-vitro studies. Moreover, it is suggested that there is a dose-response relationship between sodium arsenite concentration and the prevalence of DM [67]. Although the detailed molecular mechanism of the impact of sodium arsenite in DM is still unclear, different studies suggested pathways associated with oxidative stress and inflammation [68]. Oxidative stress induced by exposure to sodium arsenite impacts mitochondrial electron transport and non-enzymatic glycosylation reaction and consequently develops DM [69]. A recent study revealed that melatonin attenuates the devastating cardiotoxicity effects of arsenic by regulating oxidative stress-related genes (such as Nrf2), antioxidant enzymes, and apoptosis [70]. Also, oxidative stress can easily lead to impaired insulin secretion according to the lower level of antioxidant enzymes in pancreatic beta cells [[71], [72], [73]].

An in-vitro study on chronic exposure to sodium arsenite and the level of secreted insulin demonstrated that 72 h of exposure to sodium arsenite could significantly lower the expression of relevant mRNA and decrease the secretion level of glucose-stimulated insulin. This study showed that this result could be associated with intracellular calcium concentration and KATP channels, which have a crucial role in insulin secretion [72].

Moreover, sodium arsenite impaired other cellular pathways, including signal transduction factors like MAPK, PI3K, and PKB/Akt, as well as NFkB, which all have crucial roles in the uptake of glucose in adipose or skeletal tissues [[74], [75], [76]]. Phenylarsine oxide might be the intracellular derivative of sodium arsenite and inhibits glucose-stimulated insulin secretion through the translocation of Glucose transporter type 4 (GLUT4) [77].

Furthermore, in-vivo experiments on the role of sodium-arsenite in the prevalence of DM showed that exposure to sodium arsenite in rats and mice through oral gavage, addition to drinking water, and IP injection could decrease the level of liver glycogen as well as blood glucose [30,78].

MLT is newly diagnosed to have some receptors in pancreatic islets. Therefore, it can be an antioxidant agent in the inflammation and oxidative stress induced in pancreas tissue [79]. MLT receptors 1A and 1B (MTNR1A and MTNR1B), expressed in pancreatic islets, are shown to have impacts on the secretion of insulin as well as the regulation of hemostasis of glucose [79].

Some studies also suggested that nocturnal MLT secretion and its impact on insulin secretion can be confirmed through the lack of hunger during sleep [80].

Alteration of MLT levels in diabetic patients can also significantly be detected in severe neuropathic periods [81]. However, the level of insulin in DMT2 and the level of MLT in serum are demonstrated to be relevant [82]. Studies on pancreatic islets showed that MLT affects insulin secretion mainly through MTNR1B in human and rodent islets, and it is also influential in the coupling of MTNR1A and MTNR1B and controls insulin secretion through signal transduction factors [79].

Moreover, MLT's antioxidant role in pancreatic islets has been confirmed through different studies. An in-vitro study demonstrated that MLT benefits pancreatic islets in hyperglycemic conditions. MLT affects the expression of genes and proteins related to the cell cycle, such as P53, P16, and P38, all related to cellular senescence; thus, MLT was confirmed for its anti-apoptotic and anti-aging impacts [83].

Also, an in-vivo study on pancreatitis in pigs showed that MLT could significantly improve the inflammation and reaction of pancreatic islets. The results of MLT exposure were lower acinar necrosis, edema of pancreas tissue, and reduction of inflammatory cytokines [84].

Results of various studies on MLT's role in protecting pancreatic islets from the oxidative stress induced by sodium-arsenite align with the results taken from this study. MLT mainly controls the formation of oxidative stress biomarkers, prevents inflammation and alterations in gene expression, and finally improves the level of insulin secretion in pancreatic islets. In other words, MLT can be studied further to discuss its potential role in the management of DM, in particular through its pancreatic receptors.

## 5 Conclusion

In conclusion, this study was about to investigate ameliorating effects of MLT on SA-induced pancreas tissue toxicity. This study indicated that oxidative stress factors (ROS and TTM) and inflammation-related factors (such as *p53, Nrf2, TNF-α, NFkB,* and *IL-6*) which are harmful and a sign of toxicity, significantly increased due to SA exposure. This study shows that MLT administration after induction of sub-acute SA toxicity reverses these effects significantly. Also, investigations on biochemical-related factors (such as related genes including *SIRT1, Glut2, UCP2* and *Pdx-1*, blood sugar level, and insulin secretion level) in this study show that these factors are negatively altered by receiving SA. Interestingly, MLT treatment has significant potential to ameliorate these adverse effects of SA.

These findings confirmed the hypothesis that MLT has some ameliorating effects on SA-induced toxicity.

## CRediT authorship contribution statement

**Ramtin Farhadi:** Writing – original draft, Project administration, Formal analysis, Data curation, Conceptualization. **Marzieh Daniali:** Writing – review & editing, Project administration. **Maryam Baeeri:** Methodology, Formal analysis, Conceptualization. **Reza Khorasani:** Methodology. **Hamed Haghi-Aminjan:** Methodology. **Mahdi Gholami:** Project administration, Methodology. **Mahban Rahimifard:** Writing – review & editing, Formal analysis. **Mona Navaei-Nigjeh:** Formal analysis. **Mohammad Abdollahi:** Supervision, Funding acquisition, Conceptualization.

## Data availability statement

Data will be available if requested.

## Declaration of competing interest

The authors declare that they have no known competing financial interests or personal relationships that could have appeared to influence the work reported in this paper.
